# Class I Integrons and SXT Elements in El Tor Strains Isolated before and after 1992 *Vibrio cholerae* O139 Outbreak, Calcutta, India

**DOI:** 10.3201/eid0904.020317

**Published:** 2003-04

**Authors:** M. Amita, S. Roy Chowdhury, M. Thungapathra, T. Ramamurthy, G. Balakrish Nair, Amit Ghosh

**Affiliations:** * Institute of Microbial Technology, Chandigarh, India; † National Institute of Cholera and Enteric Diseases, Calcutta, India; ‡ International Centre for Diarroheal Disease Research, Dhaka 1000, Bangladesh

**Keywords:** Vibrio cholerae, drug resistance, integrons, class I, SXT, India, dispatch

## Abstract

We examined the distribution of class I integrons and SXT elements in *Vibrio cholerae* O1 El Tor strains, isolated in Calcutta, India, before and after the *V. cholerae* O139 outbreak in 1992. Class I integrons, with *aadA1* gene cassette, were detected primarily in the pre-O139 strains; the SXT element was found mainly in the post-O139 strains.

Since the introduction of antibiotics in the treatment of infectious diseases, antibiotic resistance has spread dramatically among microbes. The occurrence of drug-resistant strains of *Vibrio cholerae* is being reported with increasing frequency (*1*). Spread of antibiotic resistance in microbes has been attributed to the mobilization of drug-resistance markers by a variety of agents (e.g., plasmids, transposons, and integrons). In *V. cholerae*, antibiotic-resistance determinants have traditionally been found on plasmids. Recently, in a few cases, these determinants have also been detected on integrons and a novel conjugative transposable element, SXT.

Integrons are DNA elements capable of mobilizing individual gene cassettes into bacterial chromosomes by site-specific recombination. Integrons consist of a central variable region that often harbors antibiotic-resistance gene cassettes, flanked by 5´ and 3´ conserved sequences (CS) (*2*). Integrons have been categorized into four different classes on the basis of the distinctive integrase (*int*) genes they carry on their 5´-CS (*2*,*3*). Among the different integron families, class I integrons are found to be most prevalent in drug-resistant bacteria. Class I integrons have been detected in *V. cholerae* O1 strains isolated in Vietnam, Thailand, and Italy (*4*–*6*). Their presence in *V. cholerae* O1 strains isolated in India, however, had not been previously reported. SXT is a transmissible genetic element that harbors resistant determinants to trimethoprim, streptomycin, sulfamethoxazole, and chloramphenicol. SXT was first discovered in *V. cholerae* O139 (*7*), a new epidemic strain that emerged in the Indian subcontinent in late 1992 and displaced the *V. cholerae* O1 El Tor as the primary cholera-causing agent for approximately 6 months. After this period, *V. cholerae* O1 El Tor reemerged and became predominant (*8*). Unlike those of the pre-O139 period, most of these poststrains were found to be resistant to trimethoprim, sulfamethoxazole, and streptomycin, and in a few cases, this resistance was found to be due to the SXT element. We describe the results of a study in which we examined, retrospectively, the presence and the relative abundance of class I integrons and SXT elements in *V. cholerae* El Tor O1 strains, isolated in Calcutta, before, during, and after the O139 outbreak.

## The Study

A total of 58 strains of *V. cholerae* O1 El Tor isolated in Calcutta before (March–December 1992; group I), during (July–November 1993; group II), and after (March 1994–June 1995; group III) the *V. cholerae* O139 outbreak (*8*) were included in this study. These strains, belonging to ribotypes RI, RII, and RIII, were maintained in brain-heart infusion broth supplemented with 15% glycerol at –70°C and grown when needed (*8*).

### Occurrence of Class I Integrons

The 3´ conserved sequence of class I integrons is characterized by antibiotic resistance gene *qacEΔ1* and the sulfonamide resistance gene *sul1*. To identify class I integrons, primers ATCGCAATAGTTGGCGAAGT (accession no. X15370) and GCAAGGCGGAAACCCGCGCC (accession no. X12869), specific for 3´ CS (*5*), were amplified in this region by polymerase chain reaction as described (*5*). A 0.8-kb product, found in 22 of the 58 isolates, was confirmed to be the 3´ CS of class I integrons by sequencing (Table). To identify the gene cassette in the class I integrons, primers previously used to amplify the region between the 5´ CS and 3´ CS (*5*) were used. All 22 strains produced a 1.0-kb amplicon, which on sequencing was found to contain the *aadA1* gene that confers resistance against aminoglycosides, streptomycin, and spectinomycin (*4*,*5*). Nucleotide sequence of *aadA1* cassette has been assigned the GenBank accession no. AY115577.

**Table:** Class I integron and SXT profiles of 58 *Vibrio cholerae* strains isolated before, during, and after the 1993, *V. cholerae* O139 outbreak, Calcutta

Group	Strain no.	Ribotype^a^	Class I integron	SXT	Antibiogram^b^
I	VC1	RI	+		A, N, S, Sm
	VC2	RI	+		Fz, S, Sm
	VC3	RII			Fz, Sm
	VC5	RI	+		A, Fz, S, Sm
	VC12	RI	+		N, S, Sm
	VC14	RI		+	S, Sm, Tr
	VC20	RI	+		N, S, Tr
	VC35	RI			Fz, N
	VC41	RI			A, Fz, N
	VC44	RI	+		A, Fz, N, S, Sm
	VC48	RI	+		A, Fz, N, S, Sm
	VC54	RI	+		A, N, S, Sm
	VC55	RI	+		Fz, N, S, Sm
	VC59	RI	+		A, N, S, Sm
	VC70	RI	+		Fz, S, Sm
	VC72	RI	+		S, Sm
	VC73	RI	+		S, Sm
	VC80	RI	+		S, Sm
	VC99	RI	+	+	A, Fz, N, S, Sm, Tr
	VC100	RI	+	+	A, Fz, N, S, Sm, Tr
	VC105	RI		+	S, Sm, Tr
	VC106	RI	+		N, S
II	CO222	RI	+		Fz, S, Sm
	CO327	RIII		+	A, N, S, Sm, Tr
	CO334	RI	+	+	A, N, S, Sm, Tr
	CO366	RIII		+	A, S, Sm, Tr
	CO370	RIII		+	S, Sm, Tr
	CO371	RI	+		S, N
	CO374	RIII		+	A, N, S, Sm, Tr
	CO387	RIII		+	N, S, Sm, Tr
	CO394	RIII		+	A, Fz, S, Sm, Tr
	CO407	RIII		+	A, Fz, N, S, Sm, Tr
	CO416	RIII		+	A, N, S, Sm, Tr
	CO417	RIII		+	A, N, S, Sm, Tr
	CO423	RIII		+	A, N, S, Sm, Tr
	CO424	RIII		+	A, Fz, N, S, Sm, Tr
	CO427	RIII		+	A, Fz, N, S, Sm, Tr
III	CO458	RI	+	+	N, S, Sm, T, Tr
	CO459	RIII		+	Fz, Na, N, S, Sm, Tr
	CO460	RIII		+	Fz, Na, S, Sm, Tr
	CO461	RI	+	+	N, S, Sm, T, Tr
	CO471	RIII		+	Fz, Na, N, S, Sm, Tr
	CO473	RIII		+	Fz, Na, S, Sm, Tr
	CO474	RIII		+	Fz, Na, N, S, Sm, Tr
	CO475	RIII		+	A, Fz, S, Sm, Tr
	CO580	RIII		+	Fz, Na, N, S, Sm, Tr
	CO650	RIII		+	A, Fz, N, S, Sm, Tr
	CO720	RIII		+	Fz, Na, N, S, Sm, Tr
	CO770	RIII		+	A, Fz, Na, S, Sm, Tr
	CO810	RIII		+	Fz, Na, N, S, Sm, Tr
	CO825	RIII		+	A, Fz, Na, N, S, Sm, Tr
	CO835	RIII		+	Fz, Na, S, Sm, Tr
	CO839	RIII		+	A, Fz, Na, N, S, Sm, Tr
	CO840	RIII		+	Fz, N, S, Sm, Tr
	CO860	RIII		+	A, Fz, N, S, Sm, Tr
	CO910	RIII		+	Fz, N, S, Sm, Tr
	CO950	RIII		+	A, Fz, N, S, Sm, Tr
	CO970	RIII		+	A, Fz, Na, N, S, Sm, Tr

^a^Sharma et al. (*8*) ^b^A, ampicillin; Cf, ciprofloxacin; Fz, furazolidone; G, gentamycin; N, neomycin; Na, nalidixic acid; S, streptomycin; Sm, sulfamethizole; T, tetracycline; Tr, trimethoprim.

 A total of 17 out of the 22 class I integron-bearing strains belonged to the before period. The remaining five, though isolated after June 1993, probably represented carry-over strains. These five strains belonged to the same RI ribotype as the other 16 strains from the before period (Table) and had a CTX structure identical to the other strains with RI ribotype (*8*).

### Antibiotic Resistance Profile

Resistance of *V. cholerae* isolates to ampicillin (10 µg), ciprofloxacin (5 µg), furazolidone (50 µg), gentamycin (10 µg), neomycin (30 µg), nalidixic acid (30 µg), streptomycin (10 µg), sulfamethizole (100 µg), tetracycline (30 µg), and trimethoprim (25 µg) were examined by using commercial discs (Hi Media, Bombay, India) as described (*1*). An overall increase in the number of strains with resistance to a greater number of antibiotics was seen in the isolates of the post-O139 period; a fourfold increase in resistance against trimethoprim, a drug often used for the treatment of cholera in children and pregnant women, was noted. Almost all strains, from all isolation periods, were uniformly resistant to streptomycin and sulfamethizole, but none were found to be resistant to tetracycline, gentamycin, or ciprofloxacin. None of the strains examined were found to harbor any plasmid.

### Presence of SXT Elements

None of the strains belonging to the ribotypes RII and RIII, which were all isolated during and after the O139 outbreak, carried the *aad*A1 gene cassette (coding for aminoglycoside resistance); however, they were all resistant to streptomycin. Further, all of the strains were resistant to trimethoprim. Since strains of *V. cholerae,* which harbor the SXT element, are resistant to streptomycin and trimethoprim (*7*), we sought to determine if the resistance of the post-O139 strains to streptomycin and trimethoprim could be traced to an SXT element. Colony hybridization of the 58 strains with a 0.8-kb probe, specific for the SXT integrase gene (*7*), showed that all trimethoprim-resistant strains from all isolation periods and all during and after streptomycin-resistant carried the SXT element (Table). Further, five of these strains harbored both SXT and the *aad*A1 gene cassette (Table). Our survey thus suggested that while the streptomycin resistance of the strains isolated before the O139 outbreak was due to the *aad*A1 gene cassette carried by the class I integrons, the SXT element was probably responsible for this phenotype in the post-O139 strains.

All *V. cholerae* strains included in this study were resistant to multiple antibiotics. Since none of the strains harbored any plasmid and the resistance determinants present in the class I integrons and in the SXT element could account for only a few markers, other determinants of antibiotic resistance may exist.

Dalsgaard et al. (*4*) found that the O1 strains isolated in Vietnam in 1994 and after had the ribotype identical to that found in some of the O1 strains isolated in Samutsakorn, Thailand, during and after the 1993 O139 outbreak. Both pre- and post-O139 isolates carried class I integrons with the *aadA2* gene cassette. This fact led Dalsgaard et al. to conjecture that this distinct O1 strain might have been transferred between Thailand and Vietnam (*5*). However, the possibility of its migrating from a third country could not be ruled out. The ribotype of the pre-O139, O1 Calcutta strains examined in this study was identical to that of the Samutsakorn strains (*4*,*5*,*8*). However, as can be seen from the data presented here, unlike the Samutsakorn strains, these strains harbored *aadA1* gene cassette.

## Conclusions

We had shown previously that the post-O139, O1 strains isolated in Calcutta could have migrated to Guinea-Bissau in Africa (*9*). Further evidence has shown that this strain, which caused an outbreak in 1994–1995, could have subsequently acquired class I integrons bearing the [*ant(3”)-1a*] gene cassette by a 150-kb plasmid from an unknown source (*10*).

While the class I integron with *aadA1* gene cassette was widely distributed among the pre-O139 O1 strains isolated in Calcutta, it was mostly absent in the post-O139 O1 strains (Figure). This finding is in contrast to other studies involving the SXT element; 80% of all pre-O139 strains were devoid of SXT, whereas all post-O139 strains (with the exception of a few carry-over strains) had it (Figure). When the data presented in this paper are considered together with the information presented by others (*4*,*5*,*8*,*10*), it appears that both pre- and post-O139, O1 strains, isolated in Calcutta, probably could have moved to other countries and became established there.

**Figure:**
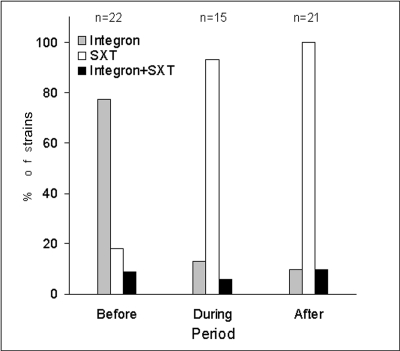
Distribution of class I integrons and SXT elements in *Vibrio cholerae* El Tor strains isolated before, during, and after the O139 outbreak.

## References

[1] Bag PK, Maiti S, Sharma C, Ghosh A, Basu A, Mitra R, Rapid spread of the new clone of *Vibrio cholerae* O1 biotype El Tor in cholera endemic areas in India. Epidemiol Infect. 1998;121:245–51. 10.1017/S0950268898001423 9825773 PMC2809519

[2] Recchia GD, Hall RM. Gene cassettes: a new class of mobile element. Microbiology. 1995;141:3015–27. 10.1099/13500872-141-12-3015 8574395

[3] Mazel D, Dychinco B, Webb VA, Davies JA. A distinctive class of integron in the *Vibrio cholerae* genome. Science. 1998;280:605–8. 10.1126/science.280.5363.605 9554855

[4] Dalsgaard A, Forslund A, Tam NV, Vinh DX, Cam PD. Cholera in Vietnam: changes in genotypes and emergence of class I integrons containing amino-glycoside resistance gene cassettes in *Vibrio cholerae* O1 strains isolated from 1979 to 1996. J Clin Microbiol. 1999;37:734–41. 9986842 10.1128/jcm.37.3.734-741.1999 PMC84539

[5] Dalsgaard A, Forslund A, Serichantalergs O, Sandvang D. Distribution and content of class I integrons in different *Vibrio cholerae* O-serotype strains isolated in Thailand. Antimicrob Agents Chemother. 2000;44:1315–21. 10.1128/AAC.44.5.1315-1321.2000 10770768 PMC89861

[6] Falbo V, Carattoli A, Tosini F, Pezzella C, Dionisi AM, Luzzi I. Antibiotic resistance conferred by a conjugative plasmid and a class I integron in *Vibrio cholerae* O1 El Tor strains isolated in Albania and Italy. Antimicrob Agents Chemother. 1999;43:693–6. 10049292 10.1128/aac.43.3.693 PMC89185

[7] Hochhut B, Waldor MK. Site-specific integration of the conjugal *Vibrio cholerae* SXT element into *prfc.* Mol Microbiol. 1999;32:99–110. 10.1046/j.1365-2958.1999.01330.x 10216863

[8] Sharma C, Nair GB, Mukhopadhyay AK, Bhattacharya SK, Ghosh RK, Ghosh A. Molecular characterization of *Vibrio cholerae* O1 biotype El Tor strains isolated between 1992 and 1995 in Calcutta, India: evidence for the emergence of a new clone of the El Tor biotype. J Infect Dis. 1997;175:1134–41. 10.1086/516453 9129077

[9] Sharma C, Ghosh A, Dalsgaard A, Forslund A, Ghosh RK, Bhattacharya SK, Molecular evidence that a distinct *Vibrio cholerae* O1 biotype El Tor strain in Calcutta may have spread to the African continent. J Clin Microbiol. 1998;36:843–4. 9508329 10.1128/jcm.36.3.843-844.1998 PMC104642

[10] Dalsgaard A, Forslund A, Petersen A, Brown DJ, Dias F, Monteiro S, Class I borne, multiple-antibiotic resistance encoded by a 150-kilobase conjugative plasmid in epidemic *Vibrio cholerae* O1 strains isolated in Guinea-Bissau. J Clin Microbiol. 2000;38:3774–9. 11015401 10.1128/jcm.38.10.3774-3779.2000 PMC87474

